# An Official Outbreak Investigation of Acute Haemorrhagic Diarrhoea in Dogs in Norway Points to *Providencia alcalifaciens* as a Likely Cause

**DOI:** 10.3390/ani11113201

**Published:** 2021-11-09

**Authors:** Hannah Joan Jørgensen, Mette Valheim, Camilla Sekse, Bjarne Asbjørn Bergsjø, Helene Wisløff, Simen Foyn Nørstebø, Ellen Skancke, Karin Lagesen, Anita Haug Haaland, Sabrina Rodriguez-Campos, Siri Kulberg Sjurseth, Merete Hofshagen, Jorun Jarp, Ole-Herman Tronerud, Gro Skøien Johannessen, Monica Heggelund, Sasja Rygg, Ellen Christensen, Mette Boye, Britt Gjerset, Morten Sandvik, Eiril Moen Soltvedt, Cecilia Wolff

**Affiliations:** 1 Norwegian Veterinary Institute, PB 64, 1431 Ås, Norway; mette.valheim@vetinst.no (M.V.); camilla.sekse@vetinst.no (C.S.); bjarne.bergsjo@vetinst.no (B.A.B.); helene.wisloff@zoetis.com (H.W.); karin.lagesen@vetinst.no (K.L.); siri.sjurseth@nortura.no (S.K.S.); merete.hofshagen@vetinst.no (M.H.); jorun.jarp@vetinst.no (J.J.); gro.johannessen@vetinst.no (G.S.J.); ellen.christensen@vetinst.no (E.C.); mette.boye@vetinst.no (M.B.); britt.gjerset@vetinst.no (B.G.); morten.sandvik@vetinst.no (M.S.); cecilia.mia.wolff@vetinst.no (C.W.); 2 Bacteriology and Mycology Unit, Faculty of Veterinary Medicine, Norwegian University of Life Sciences, PB 5003, 1432 Ås, Norway; simen.foyn.norstebo@nmbu.no (S.F.N.); sabrina.rodriguez@nmbu.no (S.R.-C.); eiril.moen.soltvedt@nmbu.no (E.M.S.); 3 University Animal Hospital, Faculty of Veterinary Medicine, Norwegian University of Life Sciences, PB 5003, 1432 Ås, Norway; ellen.skancke@nmbu.no (E.S.); anita.haug.haaland@nmbu.no (A.H.H.); 4 Norwegian Food Safety Authority, PB 383, 2381 Brumunddal, Norway; ole-herman.tronerud@mattilsynet.no; 5 Evidensia, Dronningens Gate 16, 0152 Oslo, Norway; monica.heggelund@evidensia.no; 6 Anicura Norway, Hoffsveien 70c, 0377 Oslo, Norway; sasja.rygg@anicura.no

**Keywords:** canine, diarrhoea, whole genome sequencing, haemorrhagic gastroenteritis

## Abstract

**Simple Summary:**

An official outbreak investigation to reveal the cause and possible common exposures of dogs suffering acute haemorrhagic diarrhoea (AHD) was performed by the veterinary authorities in Norway in 2019. The outbreak had been reported by private veterinarians who were consulting a greater number of dogs than usual with severe AHD. Epidemiological and diagnostic investigations pointed to the bacteria *Providencia alcalifaciens* as a possible cause of the outbreak. Whole genome sequencing of bacterial strains from 51 dogs showed that they were almost identical, which implies that the dogs had been exposed to a common source of infection. However, epidemiological investigations did not reveal a common source. Further studies are needed to investigate the disease-causing properties of *P. alcalifaciens* in dogs.

**Abstract:**

An outbreak investigation was initiated in September 2019, following a notification to the Norwegian Food Safety Authority (NFSA) of an unusually high number of dogs with acute haemorrhagic diarrhoea (AHD) in Oslo. Diagnostic testing by reporting veterinarians had not detected a cause. The official investigation sought to identify a possible common cause, the extent of the outbreak and prevent spread. Epidemiological data were collected through a survey to veterinarians and interviews with dog owners. Diagnostic investigations included necropsies and microbiological, parasitological and toxicological analysis of faecal samples and food. In total, 511 dogs with acute haemorrhagic diarrhoea were registered between 1 August and 1 October. Results indicated a common point source for affected dogs, but were inconclusive with regard to common exposures. A notable finding was that 134 of 325 faecal samples (41%) cultured positive for *Providencia alcalifaciens*. Whole genome sequencing (WGS) of 75 *P. alcalifaciens* isolates from 73 dogs revealed that strains from 51 dogs belonged to the same WGS clone. Findings point to *P. alcalifaciens* as implicated in the outbreak, but investigations are needed to reveal the pathogenic potential of *P. alcalifaciens* in dogs and its epidemiology.

## 1. Introduction

The terms acute haemorrhagic diarrhoea (AHD), AHD syndrome (AHDS), haemorrhagic gastroenteritis (HGE), and HGE syndrome have been used somewhat interchangeably to describe an acute onset of haemorrhagic diarrhoea in dogs, often accompanied by vomiting, that may progress rapidly and be fatal. HGE implies an inflammatory reaction of the gastric and intestinal mucosa, but infiltration of inflammatory cells in the intestinal mucosa may be variable and lesions in the stomach may be absent [1]. AHDS has been defined as acute-onset vomiting, anorexia, and lethargy, progressing to haematemesis, and severe malodorous diarrhoea [1]. The exact pathogenesis of the syndrome remains unknown although *Clostridium perfringens* NetE and NetF toxins may be implicated in some cases [2,3].

On the 4 September 2019, a chain of private veterinary clinics notified the Norwegian Food Safety Authority (NFSA) that they had treated an unexpectedly high number of dogs with AHD in Oslo in the preceding weeks. The Norwegian regulation on notifiable animal diseases states that an unusual distribution of diseases in animals is notifiable to NFSA [4]. The clinical picture, described as AHD, included a varying severity of acute onset of watery diarrhoea that could rapidly progress to haemorrhagic diarrhoea consistent with severe AHDS. Despite intensive care, several dogs, including young and otherwise healthy dogs, had died within 24 h or were euthanized within days due to poor prognosis. Of the veterinary hospitals in Oslo, two others, including the small animal hospital of the Norwegian University of Life Sciences (NMBU), confirmed the observation. They reported to be seeing more cases than usual of dogs with less severe acute vomiting and diarrhoea in addition to more severe cases. None of the three veterinary hospitals had detected relevant infectious or toxic agents in the sick dogs.

Following intense media coverage, public concern quickly rose that Norway could be facing an outbreak of a contagious disease among dogs. The NFSA therefore requested support from the Norwegian Veterinary Institute (NVI) to document the extent of a possible outbreak, find the cause, and give advice on how to prevent further spread. This paper describes the official outbreak investigation performed by the NFSA and the NVI with support from the NMBU and veterinarians in private veterinary clinics.

## 2. Materials and Methods

### 2.1. Case Definition

Because no specific pathogen or toxin had been detected in sick dogs, the case definition was based on clinical findings: “Dog in Norway presenting with acute haemorrhagic diarrhoea with an onset 1 August 2019 or later, and reported to NVI or NFSA by a veterinarian”. The term “acute haemorrhagic diarrhoea” (AHD) was considered to best describe the clinical picture.

### 2.2. Surveys and Interviews to Collect Epidemiological Data

To generate hypotheses on common exposures, an online survey (www.questback.com, accessed on 1 November 2021) was distributed by e-mail on the 5 September to 25 owners of dogs that fit the case description. The questions (*n* = 42) included date of onset of disease, breed, age, clinical symptoms, contacts with other dogs, postal code of household, food and treats, visited locations, and activities. Several questions were open-ended. The survey was closed 7 September and summarised.

The NVI and the Norwegian Veterinary Association distributed a second online survey (www.questback.com, accessed on 1 November 2021) to veterinarians via SMS and email by on the 8th. Veterinarians were asked to report all dogs with AHD with onset from 1 August 2019. The purpose was to systematically register cases and to collect information on clinical findings and possible risk factors. The questions (*n* = 57) included date of onset of disease, breed, age, clinical symptoms, contacts with other dogs, postal code of household, food and treats, travel, behaviour when walked (e.g., scavenging, drinking from puddles, or swimming, etc.) and visited locations (Table 1). Veterinarians were advised to complete the form together with the owner of the dog. The survey was closed 29 September and summarised. The full list of questions is available in Supplementary Table S1.

The owners of 14 dogs from which highly similar strains of *P. alcalifaciens* had been isolated and whole genome sequenced, were requested for an interview in their home by NFSA staff. A questionnaire with questions (*n* = 87) focusing on food, treats, activities and locations for walks was developed for this purpose.

### 2.3. Samples for Bacteriological Analysis

From the 8 September to the 1 October, the NVI performed bacteriological culture of faecal samples or faecal swabs from dogs with AHD free of charge, if submitted by a veterinarian.

A total of three different sets of faecal samples or faecal swabs from clinically healthy dogs with no recent history of diarrhoea or vomiting were collected. First, a convenience sample of healthy dogs living in the Oslo and surrounding area was collected between 10 and 17 September. Owners were among the staff at the NVI and NMBU. Second, healthy dogs in Bergen (*n* = 76) and Tromsø (*n* = 45) were sampled at private veterinary clinics between 4 and 16 October. Third, faecal swabs from 100 healthy dogs were made available from the programme for surveillance of antimicrobial resistance [5]. These samples for AMR-surveillance had been collected by private veterinarians all over Norway during 2019 to cover all four seasons. The swabs had been enriched in 5 mL BPW-ISO and kept frozen as enrichment stocks at −80 °C.

During home visits to owners of ten dogs with highly similar strains of *P. alcalifaciens*, samples (*n* = 16) were collected of dog food and treats that may have been given to the dogs in the week before they became ill with AHD. In addition, 12 samples of food and treats were collected by the NFSA from different households with dogs with AHD.

### 2.4. Pathology

From the 4 September, the NVI performed necropsies free of charge on dogs that had died or been euthanized due to AHD.

During the necropsy, tissue samples from stomach, and from the small and large intestine, were fixed in buffered 10% formalin for histological examination. Swabs, tissue, and intestinal content for microbiological (all necropsied dogs) and toxin analyses (five dogs) were collected from the stomach, and small and large intestine. For bacteriology, the stomach, duodenum, ileum, and colon were sampled by rolling and gently rubbing a swab over the intestinal surface to enhance the retrieval of entero-adherent bacteria. Swabs were placed in Amies transport medium with charcoal (VWR) and cultured the same day.

Formalin fixed tissue samples were embedded in paraffin, cut at 2–4 µm and stained with haematoxylin eosin (HE) and Gram-stain. In addition, tissue sections from the stomach as well as the small and large intestine from five dogs were analysed by fluorescence in situ hybridization (FISH). The FISH-analyses utilized oligonucleotide probes targeting 16S ribosomal RNA of Domain Bacterium, EUB338: 5′- GCTGCCTCCCGTAGGAGT-3′) [6], and a newly designed probe targeting nucleotide 443–460 of 16S rRNA of *P. alcalifaciens*. The species-specific probe 5′-CAACGCCTTCCTCCCAAC-3′ was designed using the software ARB (http://www.arb-home.de/, accessed on 1 November 2021). The oligonucleotide probes were purchased from LGC Biosearch Technologies, Risskov, Denmark, and were 5′ labelled with fluorescein isothiocyanate or Cy3. Hybridization was carried out at 46 °C as previously described [7]. A Nikon Eclipse 80i epifluorescence microscope equipped with a 100-W HBO lamp and a red–green double filter set MXR00201 was used to visualize the Cy3-labelled probe. Images were obtained using a Nikon DS-Ri2 camera and the software NIS-Elements D 5.02.00. Control samples for FISH were prepared by injecting pure cultures of *P. alcalifaciens* suspended in a 0.9% sterile saline solution into porcine lung samples.

### 2.5. Bacteriological Analyses

Intestinal content (stomach, duodenum, ileum, and colon) from necropsied dogs and faecal swabs or faecal samples from dogs with AHD were cultured by general bacteriological culturing. Samples from necropsied dogs were also analysed using selective methods for *Salmonella* spp. and *Campylobacter* spp. [8,9].

For general culturing, intestinal content was inoculated onto two 5% bovine blood agar (BA) plates (Oxoid, Basingstoke, UK) and a lactose, sucrose bromothymol agar (BBLS) (in-house agar). BA-plates were incubated in a 5% CO_2_ atmosphere and anaerobically, and the BBLS was incubated aerobically. All plates were incubated at 37 °C for 18–24 h. The BBLS medium was adjusted early in the outbreak to include polymyxin (250,000 U/L) to enhance the culture of *P. alcalifaciens* [10].

Samples from healthy dogs were cultured specifically for *P. alcalifacien*s on BBLS plates as described above. The 121 samples from healthy control dogs from Bergen and Tromsø were analysed at the NMBU where an additional enrichment was performed by incubating the faecal swab in 5 mL BPW-ISO at 37 °C for 18–24 h, followed by dilution (1:10) in Müller–Kauffmann Tetrathionate broth (MKTT, Merck) and incubation at 37 °C for another 18–24 h. Subsequently, 1 µL of each sample was plated onto PMXMP agar [10] and incubated aerobically at 37 °C for 18–24 h. Enrichment stocks from the 100 samples collected for AMR-surveillance were thawed at room temperature, diluted 1:10 in BPW-ISO and incubated for 3 h at 37 °C. A total of ten microliters of each sample were plated onto BBLS-agar with polymyxin as described above.

The 28 samples of dog food and snacks were cultured specifically for *P. alcalifaciens* using a modified version of [9]. Briefly, a 25 g sample was enriched with 225 mL buffered peptone water (BPW-ISO) for 18–24 h at 37 °C. If less material was available, BPW-ISO was added to obtain a 1:10 dilution. Following enrichment, 10 µL of broth was plated on BBLS-agar with polymyxin and Xylose Lysine Deoxycholate (XLD) agar (OXOID). All plates were incubated at 37 °C for 18–24 h. Aliquots of the enrichment broth were transferred to Rappaport Vassiliadis Soya (RVS, OXOID) (0.1 mL) and Müller–Kauffmann Tetrathionate Novobiocin (MKTTn, VWR) (1 mL) broth and incubated at 41.5 °C for 18–24 h. The RVS and MKTTn broths were plated onto the same solid media as described above. Suspect colonies were either identified directly using the MALDI Biotyper MS (MALDI-TOF MS, Daltonics GmbH, Bremen, Germany) or pure cultured on BBLS agar prior to identification using MALDI Biotyper MS. A score of ≥2 was considered a definitive species identification.

Of the 28 food and snack samples, twelve were also analysed quantitatively for *Enterobacteriaceae* and *Clostridium perfringens* [9]. Aliquots of the initial dilution were plated onto blood agar for the analysis of *Cl. perfringens* and Petrifilm (3M™ Petrifilm™ *Enterobacteriaceae* Count Plates) for *Enterobacteriaceae*, and incubated at 37 °C for 18–24 h. The blood plates were incubated anaerobically (Anaerocult, BioMerieux).

### 2.6. Other Microbiological and Parasitological Analyses

Samples of small intestinal content from all necropsied dogs, and faecal samples or swabs from an additional seven dogs with AHD, with a completed veterinary questback, were tested by real-time PCR at Idexx Laboratories (www.idexx.no, accessed on 1 November 2021) for Canine Parvovirus 2, Canine Enteric Coronavirus, Canine Distemper virus, Canine Circovirus, *Cl. perfringens* alpha toxin, enterotoxin and *netE/F* toxin genes, *Salmonella* spp.*, Giardia* spp. and *Cryptosporidium* spp.

Samples of intestinal tissue and content, and spleen tissue from the first four necropsied dogs were tested for *Francisella tularensis* by real-time PCR [11]. Samples from small intestinal tissue and intestinal content from the same four dogs were also tested by PCR for the genes *stx*_1_, *stx*_2_ and *eae* to detect Shiga toxin-producing *E. coli* (STEC) as described elsewhere [12]. The latter analysis was performed because during the autumn of 2019 there was an ongoing outbreak of STEC among humans in Norway. Kidney tissue samples from four dogs were sent to the Swedish Veterinary Institute for detection of pathogenic *Leptospira* spp. by real-time PCR.

Stomach content and small intestinal content from six necropsied dogs, and faecal samples from four arbitrarily selected dogs with AHD from which faecal samples were submitted to the NVI, were cultured for yeasts and moulds. Stomach content and small intestinal content were inoculated on Sabouraud Dextrose Agar (SAB, Difco, fisher scientific, Oslo, Norway) and Malt Extract Agar (MEA, Oxoid, Oslo, Norway) for seven days at 30 °C and 37 °C. Stomach content was also inoculated on Dichloran 18% glycerol Agar (DG18, Oxoid) at 25 °C. Faecal samples were inoculated on SAB agar for two days at 37 °C.

Parasitological examination of intestinal content from necropsied dogs with regard to parasites, eggs and oocysts was performed using direct microscopy in sucrose and lugol, as well as Immune Fluorescent Antigen Testing for *Cryptosporidium* spp. and *Giardia* spp. using Crypt-a-Glo and Giardi-a-Glo kits, respectively (Waterborne, INC., New Orleans, LA, USA).

### 2.7. Analyses for Toxins

Extracts from kidney tissue samples from four dogs were analysed by liquid chromatography, mass spectrometry instrument (LC-MS) to test for harmful chemicals including melamine (1,3,5-Triazine-2,4,6-triamine), glyphosate (*N*-(phosphonomethyl)glycine—Roundup), *N*-nitrosoglyphosate (*N*-Nitroso-*N*-(phosphonomethyl)glycine) and Fipronil ((RS)-5-Amino-1-[2,6-dichloro-4-(trifluoromethyl)phenyl]-4-(trifluoromethylsulfinyl)pyrazole-3-carbonitrile).

### 2.8. Pulsed-Field Gel Electrophoresis (PFGE) of P. alcalifaciens

PFGE was carried out using a modified protocol for *E. coli* [13] using *Sma*I restriction enzyme on 16 *P. alcalifaciens* isolates from 16 dogs related to the outbreak, and a *P. alcalifaciens* reference strain (DSMZ 30120). *Salmonella* Braenderup H9812 digested with *Xba*I was used as a standard. Similarity of banding patterns was evaluated manually.

### 2.9. Whole Genome Sequencing of P. alcalifaciens

DNA was extracted using QIAamp DNA mini kit (Qiagen, Germantown, MD, USA) and sequenced using Nextera Flex library prep (Illumina, San Diego, CA, USA) followed by sequencing on MiSeq (Illumina) at the Norwegian Sequencing Centre, resulting in 300 bp paired-end reads.

Initial quality control and assembly of isolates was performed using an in-house Nextflow Bifrost pipeline [14]. Briefly, this pipeline consists of reading the quality control, quality and adapter trimming, removal of PhiX and assembly. The phylogenetic analyses were performed using ParSNP [15], Gubbins [16] and IQTree [17]. The phylogenetic analysis revealed a subcluster of closely related isolates. Further analysis of the multiple alignment that ParSNP produced was performed to investigate the number of single nucleotide polymorphisms (SNPs) differences between isolates.

### 2.10. Trace-Back of Food and Treats

The NFSA analysed data on foods and treats from the veterinary survey for all dogs that were positive for *P. alcalifaciens*, and for dogs whose owners were interviewed by the NFSA. The number of dogs that had been given the same food or treat, including different brands from the same producer, was summarized.

## 3. Results

### 3.1. Description of the Outbreak

In the preliminary hypothesis-generating survey sent to 25 dog owners to reveal common exposures among sick dogs, we received 22 replies. Results were inconclusive with respect to common exposures.

In the subsequent veterinary survey, to reveal common exposures and register cases, we received 233 responses. In total, 207 dogs reported by 90 different veterinary practices met the case definition. Not all respondents answered all the questions. Therefore, the number of case dogs with replies varies between questions in the following descriptions.

**Figure 1:**
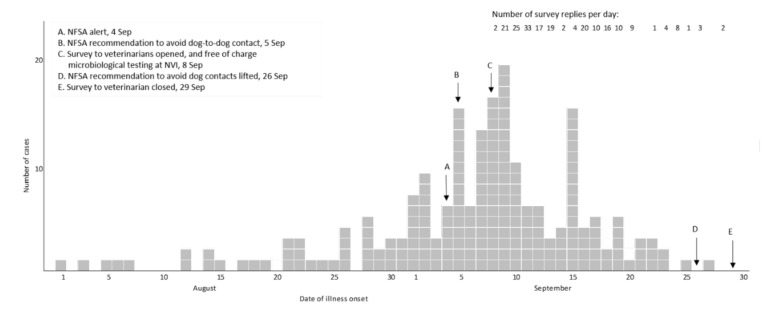
Day of illness onset for cases (*n* = 207) of acute haemorrhagic diarrhoea in dogs, and number of replies per day, in online survey to veterinarians, Norway 2019.

**Figure 2:**
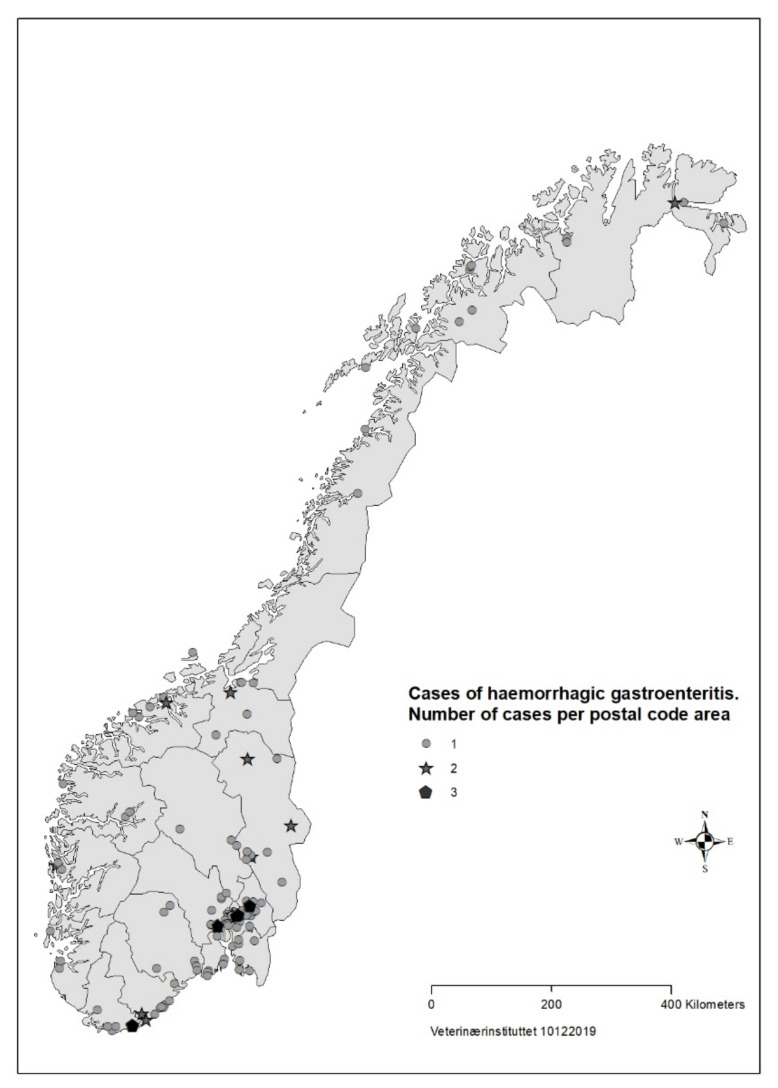
Geographical distribution of cases (*n* = 207) with acute haemorrhagic diarrhoea in dogs with onset from 1 August to 30 September reported in survey to veterinarians, Norway 2019.

For 183 (88%) of the cases the date of illness onset was between 26 August and 23 September (Figure 1). The number of responses per day dropped in the third week of the survey. Cases were reported from all counties of Norway, but mainly from Oslo and south-eastern Norway (Figure 2).

The dogs were evenly distributed with respect to age, ranging from 11 weeks to 16 years, with a mean of six years and a median of five years. In total, 87 breeds were represented, and the most frequent were mixed breed (*n* = 27), English Setter (*n* = 11), Yorkshire Terrier (*n* = 8), Golden Retriever (*n* = 7), and six each for Tibetan Spaniel, Shetland Sheepdog, and Chihuahua. In addition to haemorrhagic diarrhoea, symptoms included vomit without blood in 126 (61%), vomit with blood in 26 (13%), and fever in 23 (11%) dogs. At the time of reporting, 104 (52%) dogs had recovered or had returned home, 71 dogs (34%) were still ill, and 22 dogs (11%) were euthanized or had died before or after veterinary treatment.

In 45 of 62 (73%) households with >1 dog, only one dog had GI symptoms at the time of reporting. A total of twenty-two of 200 (11%) sick dogs had been in contact with another dog with diarrhoea in the two weeks before disease onset. A wide range of brands and types of food and treats were described for the registered dogs. The most common food was dry food, which 184/199 (92%) dogs had more or less every day (Table 2). Out of 204, sixty-three (31%) stated that the dog had changed food in the last month.

Dogs that were up to date with respect to vaccination with core-vaccines, meaning vaccinated or revaccinated within the last three years, were 174 (84%) for Canine Parvovirus, 173 (84%) HCC, and 172 (83%) for canine distemper, respectively. For the remaining dogs, 73% respondents stated that the dog had been fully vaccinated as a puppy. Hence, 94% of the dogs had been vaccinated with core vaccines at some point in their life.

Out of 207, thirty-two dogs (15%) were reported to have a concurrent medical condition. A total of twenty-two (11%) dogs had been abroad in the last two months, and 19 of these had been to Sweden. The dogs had mainly been walked in the neighbourhood, a forest, countryside, or in or near a populated area (Table 3). Of the dogs, two-thirds had drunk water from a creek or had eaten something outdoors (Table 4). From the free-text descriptions of locations and activities, a large number of parks, recreational areas, beaches, and rivers, etc. were described. None stood out as common.

The NVI received faecal samples from 371 diseased dogs altogether for bacteriological analysis. From descriptions in referral notes, 325 of these dogs were defined as having AHD, and 58 of these were identified among the 207 cases in the veterinary survey. Clinical findings of remaining dogs were not described on the referral notes. In addition, from 5 to 9 September the NFSA and NVI received notifications (email or phone call) regarding 37 dogs that met the case definition, but where no faecal sample was received at the NVI lab and no report was made in the veterinary survey. Thus, the total number of reported cases was 511.

### 3.2. Identifying the Cause of the Outbreak

#### 3.2.1. Pathology

A total of eighteen dogs were received at the NVI for necropsy between the 2 and the 25 September. A total of two had died acutely, while 16 had received veterinary care (e.g., fluid treatment and antibiotics).

**Figure 3:**
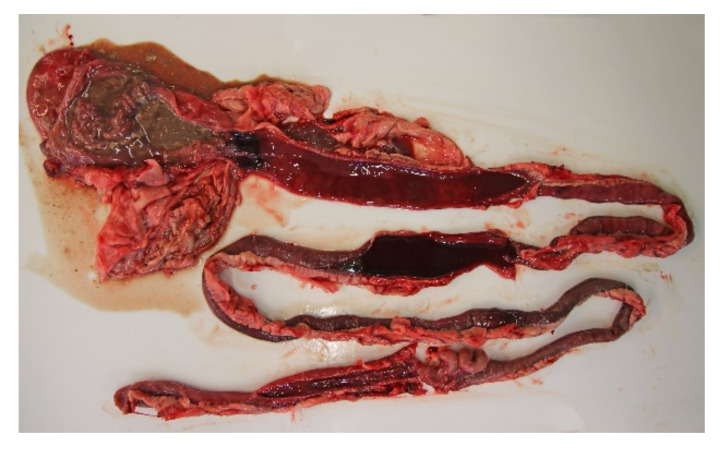
Gastrointestinal tract from a dog with acute haemorrhagic diarrhoea. The stomach content was brown and watery, and there was brown-red discoloration of the gastric mucosa. In both the small and large intestine, the mucosa was dark red and there was sparse blood-stained fluid content.

All of the 18 necropsied dogs were in normal or above average nutritional status with a weight range of 2.1 kg to 27 kg, and mean and median weights of 9.3 kg and 10.7 kg, respectively. A total of seven dogs were moderately to severely dehydrated, and several dogs had blood-stained faeces in the perineal coat. Most dogs had a brown and watery stomach content, and a brown-red discoloration of the gastric mucosa. The small intestinal content was fluid and deeply red blood-stained (Figure 3). The content of the large intestines was sparse, but likewise fluid and dark red. The intestinal mucosa was brown or dark red to violet, smooth and glossy, and in most cases the changes involved the whole intestinal tract.

The histological examination of gastric mucosa showed only minor changes in the epithelium in the majority of the dogs. However, there were varying amounts of bacteria in Gram-stained sections, with both small Gram-negative rods and large Gram-positive rods on the surface and in the glands.

**Figure 4:**
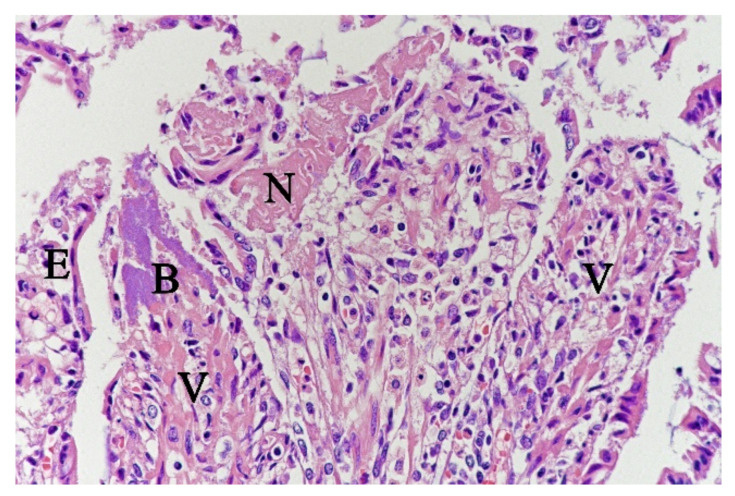
Histological section from small intestine dog with acute haemorrhagic diarrhoea, HE-stain. There was a loss of surface epithelium, necrotic epithelial cells (N), and elongated/stretched epithelial cells (E) trying to cover *lamina propria*. Note the abundance of bacteria (B) in a necrotic area on one of the villi (V).

**Figure 5:**
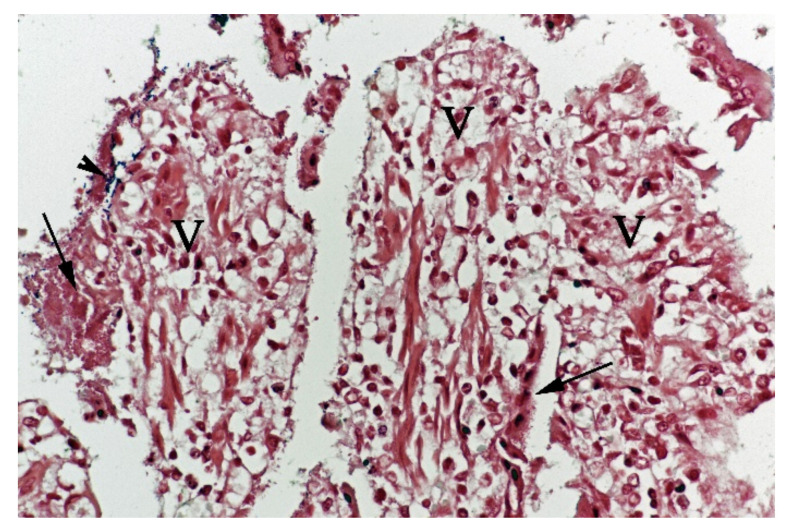
Histological section from small intestine dog with acute haemorrhagic diarrhoea, Gram-stain. Large amounts of small Gram-negative rods (arrows) were located as single bacteria or thick layers close to the surface of the villi (V). Small amounts of large Gram-positive rods (arrowhead) were present in the clusters of Gram-negative bacteria.

In contrast, the small intestinal mucosa showed dramatic changes with diffuse loss of surface epithelium and loss of architecture with collapse of the *villi lamina propria*. In some of the dogs, necrosis of intestinal epithelial cells was observed, and multifocal haemorrhages were seen in the deep *lamina propria* and submucosa (Figure 4). Gram-stained sections showed rich amounts of small Gram-negative rods, located as single bacteria or thick layers close to the surface or as larger clusters deeper in the collapsed *lamina propria* (Figure 5). Additionally, varying, but often rich amounts of large Gram-positive rods often adherent to the denuded or naked remnants of the *villi lamina propria*, were observed. The histopathological changes in the large intestine were similar to the findings in the small intestine, with the observation of the same types of bacteria on the surface and in the *lamina propria*.

**Figure 6:**
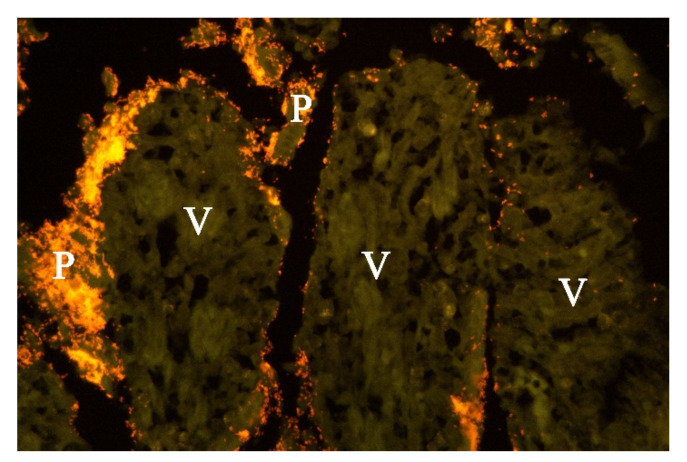
Histological section from small intestine of dog with acute haemorrhagic diarrhoea (a serial section to Figure 5. *Providencia alicafaciens* was visualised by in situ hybridization using a Cy3 labelled probe. The tissue is orange due to autofluorescence using a red–green double filter set. The Gram-negative bacteria shown in Figure 5 were identified as *P. alcalifaciens* (P). V = villi.

Examination of FISH labelled sections from the stomach, small and large intestine from five of the dogs from which *Providencia alcalifaciens* had been cultivated, showed that multiple single bacteria and large clusters of small Gram-negative rods were identified as *P. alcalifaciens* in all examined sections, corresponding to the locations described above for the Gram-negative rods (Figure 6).

#### 3.2.2. Microbiological Results

Of the 18 necropsied dogs, samples from 16 of these were positive for *P. alcalifaciens* by culturing (MALDI-TOF scores 2.2–2.36). Of these, eight were also positive for *Clostridium perfringens*, two were also positive for *Campylobacter jejuni* and one for *C. upsaliensis*.

Altogether 134 of 325 (41%) of faecal samples or swabs from dogs with AHD received at the NVI between the 4 September and 1 October were culture-positive for *P. alcalifaciens,* 138 (43%) of the dogs were culture-positive for *Cl. perfringens*, and 45 (14%) were positive for both *Cl. perfringens* and *P. alcalifaciens.* A total of three dogs (all necropsied) were culture positive for *Campylobacter* spp. No dogs were positive for *Salmonella* spp.

Faecal samples and swabs were also collected from healthy dogs to test for *P. alcalifaciens.* A total of four of 37 (11%) samples from clinically healthy dogs from Oslo cultured positive for *P. alcalifaciens*. A total of two of the 121 (1.7%) faecal samples from clinically healthy dogs sampled in Bergen and Tromsø cultured positive for *P. alcalifaciens.* None of the 100 faecal samples (stock samples) from healthy dogs collected during 2019 for AMR-surveillance, were positive for *P. alcalifaciens*.

Faecal samples from three out of 25 dogs submitted for real-time PCR-analyses were positive for Parvovirus, but two of these were identified as an attenuated vaccine virus strain. A total of two samples from two dogs were positive for canine circovirus, 15 samples were positive for *Cl. perfringens* alpha toxin gene and six were positive for *Cl. perfringens* enterotoxin gene. A single dog was positive for genes encoding *Cl. perfringens* toxins (*NetE*/*NetF*). PCR-tests for other pathogens were negative.

None of the samples tested were positive for yeasts or mould fungus, *F. tularensis*, STEC, *Leptospira* spp., *Giardia* spp., or *Cryptosporidium* spp., or for harmful chemicals.

None of the 28 samples of food or treats collected from the homes of sick dogs were positive for *P. alcalifaciens.* The numbers of *Enterobacteriaceae* and *Cl. perfringens* were below the detection level (<10 cfu/g and <100 cfu/g, respectively) in the 12 samples that were analysed for these parameters.

#### 3.2.3. Genotyping of *P. alcalifaciens*

A total of sixteen *P. alcalifaciens* isolates from 16 dogs were PFGE-typed, and ten of the isolates from ten different dogs had an identical banding pattern. The PFGE results were supported by the phylogenetic analysis from the WGS data.

In total, 79 *P. alcalifaciens* isolates from 77 dogs were whole genome sequenced, including four from clinically healthy dogs. A total of two dogs were represented by two isolates each, where one isolate was collected early in the disease course and the other post-mortem at necropsy.

SNP-based phylogenetic analysis of whole genome sequences was performed. A total of two isolates from two diseased dogs were removed from the analysis because they had a considerably greater SNP difference to the other isolates, to the extent that they were unlikely to be associated with the outbreak. When these isolates were included, the high SNP differences lead to such long branches in the phylogenetic analyses that results were difficult to interpret.

**Figure 7:**
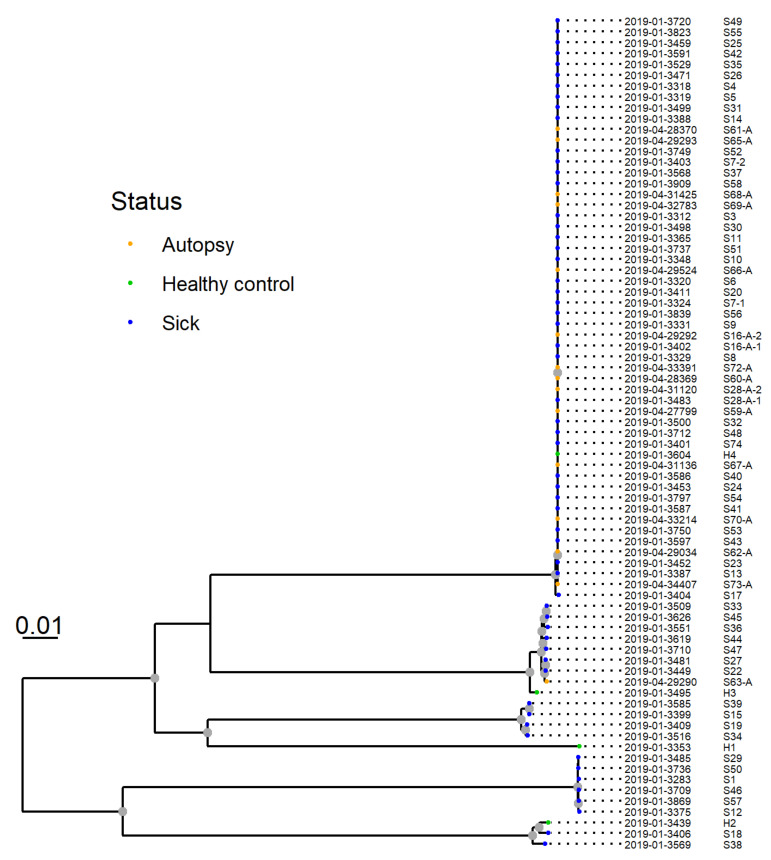
SNP-based phylogeny of whole genome sequences from 77 *Providencia alcalifaciens* isolates from 75 dogs. *P. alcalifaciens* from sick and diseased dogs (*n* = 53), including one isolate from a healthy dog, clustered closely together. This cluster was designated “clone A”, and shared 81.1% of their genomes and had six or fewer SNP differences.

The resulting phylogeny for 77 isolates from 75 dogs revealed that 53 of the isolates from 51 dogs clustered closely together. This group was designated “Clone A” (Figure 7). The two pairs of isolates from the two dogs sampled twice were both within this cluster and clustered together.

The multiple alignment that formed the basis for the tree covered an average of 81.1% of the sequenced genomes. Hence, 81.1% of the genome sequences were sufficiently conserved between the isolates so that they could be considered homologous, and thus similar enough that SNPs could safely be discerned. Analysis of this alignment showed that the clonal isolates differed by six or fewer SNPs. Other methods based on WGS were also applied, such as gene-by-gene comparison and the same clustering structure was identified (results not shown).

#### 3.2.4. Commonalities between Dogs with the Same *P. alcalifaciens* Sequence Variant

The NVI received a faecal sample or faecal swab from 58 of the 207 dogs reported in the veterinary survey. Of these, forty-nine (85%) were positive for *P. alcalifaciens,* and 46 of the isolates were whole genome sequenced. Isolates from 38 of these dogs belonged to “Clone A”. The reported clinical symptoms for these 38 dogs, in addition to haemorrhagic diarrhoea, included vomit without blood in 23 (61%), vomit with blood in 5 (13%), and fever in one dog (3%). The disease onset date ranged from 28 August to 16 September. All thirty-eight of the dogs were from Oslo and south-eastern Norway, with addresses from at least 33 different postal codes. The 38 dogs included 26 breeds, and age was evenly distributed from <0.5 to 15 years old. A total of seventeen (45%) had changed food the last few weeks before disease onset.

Of the four isolates from the four *P. alcalifaciens*-positive healthy dogs*,* one isolate was Clone A. This dog had visited the facilities of a veterinary clinic in Oslo, but had no known direct contact with sick dogs.

#### 3.2.5. Interviews with Dog Owners and Trace-Back of Food

Of the 14 owners with a case dog positive for WGS Clone A that were contacted for a home-visit of and in-depth interview by the NFSA, ten accepted the offer. The interviews revealed no common food- or treat-products, contacts or visited locations between the 10 dogs.

Similarly, no food or treats were identified as common among cases reported in the survey. This also remained the case when data from dogs that were positive for *P. alcalifaciens*, and data from dogs that were positive for *P. alcalifaciens* Clone A, were analysed separately. None of the 16 food samples collected at dog owner interviews were positive for *P. alcalifaciens*.

## 4. Discussion

We describe the first major investigation of a disease outbreak of AHD of unknown cause in dogs in Norway, where a broad diagnostic approach was coupled with extensive epidemiological investigations. Altogether, 511 different dogs with AHD were registered or sampled during the outbreak between the 1 August and 1 October 2019. Our findings point to the implication of *P. alcalifaciens,* but a common source of exposure was not identified.

The cases all had AHD, with symptoms ranging from mild diarrhoea with blood streaks to profuse and fatal haemorrhagic diarrhoea. There are many possible causes of AHD, so although the case definition was sensitive it lacked specificity, and the 511 cases most likely included the normal background incidence of AHD. An underreporting of mild cases is also likely.

An increase in incidence of AHD during the outbreak could not be documented because the background incidence of AHD in dogs in Norway is unknown. A total of two veterinary clinic chains and one small animal hospital provided data to show that they had treated a higher number of diarrhoea cases in dogs in August 2019 compared to August 2018 before media coverage started (data not shown). This was most obvious for the veterinary clinics located in the eastern and central regions of Norway. Syndromic surveillance of disease in companion animals could have provided baseline information, but is presently not performed in Norway.

Norwegian authorities quote an estimate of 560,000 dogs in Norway [18], but no data on their geographic distribution. Without such data, it was not possible to evaluate the case distribution relative to dog-population density, so the investigation focussed on documenting the temporal and spatial distribution of cases. The shape of the epicurve suggested a common point source, although a reporting bias following intense media coverage cannot be ruled out. If the incidence of AHD had continued to increase or plateau, however, the number of replies to the survey and of samples received at the NVI laboratory is unlikely to have decreased as markedly.

No age group of dogs appeared to be at a higher risk, and the breeds with the highest number of affected dogs are all common in Norway. Small or miniature dogs were not over-represented, but because case registrations did not include weight, the large group of mixed breed dogs were not categorised with respect to size.

On the 5 September, the NFSA issued a recommendation to keep dogs on the leash, avoid contact with other dogs and keep dogs with gastrointestinal symptoms isolated, but any possible effects of this intervention could not be evaluated from the collected data.

The culturing of *P. alcalifaciens,* in almost pure culture, from intestinal content from the first three necropsied dogs early in the outbreak was striking. Throughout the investigation, *P. alcalifaciens* was detected in 42% (*n* = 134) of the samples from dogs with AHD. With respect to healthy dogs sampled during the outbreak, 11% of the sampled dogs from Oslo were positive for *P. alcalifaciens* while only 2% of samples from Bergen and Tromsø were positive. *P. alcalifaciens* was not detected in any of the 100 samples from healthy dogs collected as part of the Norm-Vet surveillance during 2019.

In our outbreak investigation, *P. alcalifaciens* frequently grew in an almost pure culture on the BBLS plates. Increased awareness of *P. alcalifaciens* may have enhanced sensitivity of detection during the outbreak, but it is unlikely that the laboratory would have missed similar outbreaks in previous years. In fact, *P. alcalifaciens* was previously proposed as the cause of diarrhoea in six Norwegian dogs in 2005 [19] and five dogs in 2006 (personal communication, Trine L’Abée-Lund, NMBU). At the NVI, *P. alcalifaciens* was the main finding in canine diarrhoeal samples only four times between 2006 and 2019, despite analyses of several thousand samples (results not shown).

Other authors have also associated *P. alcalifaciens* with enteritis in dogs [20,21], while some have not considered it as a cause of illness [22]. *P. alcalifaciens* may cause pneumonia in piglets [23], and food-borne outbreaks of gastrointestinal illness in humans [24,25,26,27]. It has also been associated with diarrhoea in children [28,29], and travellers’ diarrhoea [10,30]. In our outbreak investigation, fluorescent in situ hybridisation, demonstrated *P. alcalifaciens* in large clusters deep in collapsed *lamina propria* of the intestines and adherent to the denuded remnants of the *villi lamina propria*. Altogether, we believe that the results indicate a pathological role of *P. alcalifaciens* in affected dogs.

No pathogens or toxins other than *P. alcalifaciens* were identified as a likely and common cause of disease in the case dogs. The possible exception was *Cl. Perfringens,* which grew in 43% of the faecal samples. *Cl. perfringens* has a ubiquitous environmental distribution, is part of the normal microbiota of humans and animals, and is a common finding in dogs with AHD [1], but is generally thought to cause less severe illness [31]. The role of *Cl. perfringens* alphatoxin alone in causing enteritis is controversial [32,33], but strains with the pore-forming toxins NetE/NetF have been associated with severe AHDS [2,34]. In our investigation, only one of 25 samples submitted for toxin gene quantification tested positive for *netE/netF*. It is possible that *Cl. perfringens* can lead to more severe diseases in co-infection with *P. alcalifaciens*. Further investigations of *Cl. perfringens* with respect to toxin type and pathogenicity in co-infection with *P. alcalifaciens* will be studied in an ongoing project.

SNP-based phylogenetic analysis of whole genome sequences revealed that many, but not all, of the *P. alcalifaciens* isolates clustered closely together. The *P. alcalifaciens* isolates (*n* = 53) from 51 dogs belonged to “clone A” and only differed by six or fewer SNPs. A low number of SNPs and bootstrap support for the cluster, as observed in our analyses, are among the main criteria that have been suggested as a guideline on how to interpret WGS-based phylogenies in the context of foodborne outbreaks [35]. Our findings demonstrate that *P. alcalifaciens* from 51 dogs are so similar that they could originate from a common source.

Analyses of survey data could not identify a common exposure in the case dogs, not even with more stringent case definitions. First, by adding the detection of *P. alcalifaciens* as a criterion, and then the detection of *P. alcalifaciens* clone A. A final attempt involving home visits and extended interviews of 10 owners of dogs with *P. alcalifaciens* clone A did not provide any answers either. On these grounds it was not surprising that none of the 28 treat or food samples were positive for *P. alcalifaciens*.

It is possible that *P. alcalifaciens* has an environmental source. It has been isolated from coastal water in Malaysia [36], but an occurrence in the Norwegian environment has not been investigated. The outbreak notification to NFSA came after a period of heavy rain with measurements of 42.8% and 87.6% above average rainfall for August and September 2019, respectively [37]. It also coincided with the end of the annual ban for dogs to be off the leash on the 20 August. A total of two-thirds of the dogs had drunk water from a creek or scavenged, which would agree with a *P. alcalifaciens* source in the environment. Scavenging is a known risk factor for diarrhoea in dogs [38,39].

Based on data from the survey, we concluded that the disease was not highly transmissible between dogs. The majority of households with >1 dog only had one sick dog, and there was mostly only one reported case per postal code (Figure 2). About one third of cases in the survey had recently had a food change, another risk factor for diarrhoea in dogs [39].

No more than 15 dogs with *P. alcalifaciens* as a possible cause of diarrhoea were registered in Norway by the NVI and NMBU before the outbreak in 2019. All of these cases occurred in the autumn between August and November (data not shown). Between December 2019 and August 2020, no canine faecal samples were positive for *P. alcalifaciens* at the NVI-laboratory, despite a high awareness of this potential pathogen. However, a few cases were registered in August and September 2020 (result not shown). It is possible that *P. alcalifaciens* is involved in a seasonal diarrhoea in dogs.

## 5. Conclusions

We describe the first major outbreak investigation in Norway of a disease in dogs. Obtaining an overview of the extent of the outbreak was challenging because of the lack of dog population data and poor availability of baseline disease incidence data. Based on current animal health regulations, some public financing of the outbreak investigation was possible, which was a benefit. Pathological, microbiological, and whole genome sequencing results indicate that *P. alcalifaciens* did contribute to disease in this outbreak. However, despite the fact that the temporal and spatial distribution of cases was suggestive of a common point source, the epidemiological analyses did not identify a source or common risk factors. The limited knowledge on *P. alcalifaciens* as a pathogen in dogs, including possible sources of infection, incubation period and pathogenesis precludes firm conclusions. Further research to investigate the role of *P. alcalifaciens* in AHD in dogs and to reveal relevant sources of infection is needed.

## Figures and Tables

**Table 1:** Summary of questions in an online survey to veterinarians with dog patients with acute haemorrhagic diarrhoea (AHD) in dogs, Norway, September 2019.

Category	Parameters (All Categories Had Several Free Text Fields for Comments and Details)	Exposures during the Period before Illness Onset
The dog	Age, breed, sex, vaccination status, other diseases, any medical treatments (e.g., deworming, antibiotics, NSAID, vitamins or supplements)	Other treatments: the last two months
Course of illness	Date of symptom onset, duration of illness, outcome: recovered, still ill, dead, or euthanized before or after receiving treatment	
Clinical symptoms	General appearance, inappetence, diarrhoea, diarrhoea with blood, fever, retching, drooling, vomiting with blood, vomiting without blood, bleeding other than from the intestine, tympanic abdomen, seizures, respiratory symptoms, confusion, unconsciousness, any other symptoms, and for each of these if observed by the owner and/or the veterinarian	
The dog’s household	Number of dogs, number of dogs with gastrointestinal symptoms, contacts with any other dogs with diarrhoea, postal code and postal area of the owner, other animals in the household such as cats, rodents, birds, horses, cattle, and if any of the other animals had gastrointestinal symptoms	Other dogs with diarrhoea in the last two weeks before disease onset
Activities	Places the dog had visited or been walked in, and whether on a lead or free to roam. If it had swam a lake, river, or the sea, and if it had been scavenging. If the dog had visited another country or municipality, participated in dog shows, been used for hunting, visited a veterinarian or kennel, if it had found and eaten something outdoors	Visit abroad in the last two months. Other activities in the last two weeks
Food	Any change of food. How often the dog is given dry food, wet food, and different treats such as chewing bones, products from cattle or swine skin, swine ears, snack bites pre-packaged or from a “pick and mix bar”	Change of food in the last month. How often the dog was fed foods and treats in the last two weeks
Analyses	Diagnostic laboratory tests (e.g., microbiology and toxicology). If yes, provide information on results.	
Treatments of the HGE	Outpatient or hospitalized, fluid therapy, antimicrobials, probiotics, diet	
Contact details	To the reporting veterinarian, and with permission, also to the owner	

**Table 2:** Description of different foods and treats given to registered case dogs in an outbreak of acute haemorrhagic diarrhoea. Online survey to veterinarians, Norway, September 2019.

	More or Less Daily		Some Days		Maybe		Not Given		Do Not Remember		
	*n*	%	*n*	%	*n*	%	*n*	%	*n*	%	Sum
Dry commercial food	184	92	5	3	2	1	5	3	3	2	199
Treats, prepacked	35	18	60	32	7	4	73	38	15	8	190
Wet food, prepacked	30	16	24	13	8	4	120	63	7	4	189
DentaStix™ or similar	28	14	46	24	6	3	104	53	11	6	195
Dried meat	11	6	41	21	9	5	121	62	12	6	194
Dog sausage	7	4	10	5	8	4	157	83	8	4	190
Rumen (raw food)	7	4	10	5	3	2	163	85	8	4	191
Pork treats e.g., dried ears	4	2	36	19	7	4	135	70	11	6	193
Raw food (other than rumen)	4	2	5	3	2	1	174	92	5	3	190
Beef treats e.g., dried hide	3	2	23	12	11	6	146	75	11	6	194
Treats, snacks, “pick and mix”	1	1	11	6	5	3	160	84	14	7	191

**Table 3:** Description of where case dogs were walked the 14 days before illness onset. Online survey to veterinarians with dog patients with acute haemorrhagic diarrhoea in dogs, Norway, September 2019.

Place for Walks	*n*	%
Local neighbourhood	184	89
Forest, countryside	101	49
In or near a populated area	89	43
Different municipality(ies) from home	66	32
Park	68	33
By a river	46	22
In the mountains	45	22
By a lake	36	17
Beach by the sea	33	16
A farm with animals	24	12
Dog park	22	11
Total	207	

**Table 4:** Description of activities and places visited for case dogs the 14 days before illness onset. Online survey to veterinarians with dog patients with acute haemorrhagic diarrhoea in dogs, Norway, September 2019.

Activity	*n*	%
Drank water from a creek	100	67
Ate something found outdoors (scavenging)	89	60
Ate animal faeces	37	25
Visited a veterinarian	32	21
Swam in a lake	32	21
Swam in a river	30	20
Swam in the sea	17	11
Dog show	14	9
Hunting	8	5
Ate human faeces	5	3
Stayed in a kennel	4	3
Total	149	

## Data Availability

The genome data will be submitted to ENA.

## Supplementary Materials

The following are available online at https://www.mdpi.com/article/10.3390/ani11113201/s1, Supplementary Table S1: Veterinary survey questions.
