# Supplementary material for: An Official Outbreak Investigation of Acute Haemorrhagic Diarrhoea in Dogs in Norway Points to Providencia alcalifaciens as a Likely Cause
Source: Animals (Basel). 2021 Nov 9;11(11):3201. doi: 10.3390/ani11113201 (PMC8614335; doi:10.3390/ani11113201)
Supplement: Supplementary file 1 [file animals-11-03201-s001.zip › animals-1444663-supplementary.pdf]

Providencia alcalifaciens as a possible cause of an outbreak of acute haemorrhagic diarrhoea in dogs in Norway, 2019

Jørgensen Hannah Joan[1*], Valheim Mette[1], Sekse Camilla[1], Bergsjø Bjarne Asbjørn[1], Wisløff Helene[1], Skancke Ellen[2], Simen Foyn Nørstebø[3], Karin Lagesen [1], Haaland Anita Haug[2], Sabrina Rodriguez-Campos[3], Sjurseth Siri Kulberg[1], Hofshagen Merete[1], Jarp Jorun[1], Tronerud Ole-Herman[4], Boye Mette[1], Johannessen Gro Skøien[1], Heggelund Monica[5], Rygg Sasja[6], Christensen Ellen[1], Gjerset Britt[1], Sandvik Morten[1], Wolff Cecilia[1]

1) Norwegian Veterinary Institute, PB 750 Sentrum, Oslo N-0106, Norway

2) University Animal Hospital, Faculty of Veterinary Medicine, Norwegian University of Life Sciences , Ullevålsveien 72, N-0454 Oslo, Norway

3) Bacteriology and Mycology Unit, Faculty of Veterinary Medicine, Norwegian University of Life Sciences, Ullevålsveien 72, N-0454 Oslo, Norway

4) Norwegian Food Safety Authority, Postboks 383, N-2381 Brummundal, Norway

5) Evidensia, Dronningens gate 16, N-0152 Oslo, Norway

6) Anicura Norway, Hoffsveien 70c, N-0377 Oslo, Norway

Information text about the questionnaire: This questionnaire is sent on behalf of the Norwegian Food Safety Authority. It is designed by the Norwegian Veterinary Insitute and the Norwegian University Of Life Sciences, Faculty of Veterinary Medicine. The purpose is to record cases of dogs with haemorrhagic diarrhoea to investigate possible commonalities and case distribution. We ask veterinarians to register dogs with acute haemorrhagic diarrhoea with disease onset from the 1st August 2019. It is possible to register cases retrospectively. To register more cases click the URL-link again.

**Question number**	**Question**	**Alternative answers/fields to be completed**
	Responsible veterinarian	Veterinary number/registration
		Veterinary hospital/clinic
		E-mail address
		Telephone
	Owner information (with owner's concent)	E-mail address
		Telephone
	Complete a seperate form for every sick dog in the household)	
	Information about the dog	Name
		Breed
		Age
		Postal code where the dog lives
		Chip/ID
	Sex	Male
		Female
		Neutered
		Not neutered
	Is the dog otherwise healthy	Yes
		No
		I don't know
	Underlying diseases	Describe
	Disease onset	Date (dd.mm.yy)
	Outcome of disease	The dog recovered
		The dog is still ill
		The dog died despite veterinary treatment
		The dog died before it was brought to a veterinarian
		Other (describe)
	For how long was the dog sick before it recovered or died?	Less than one day
		1-2 days
		3-4 days
		More than 4 days
	What symptoms did the dog have	Fever
		Somewhat depressed
		Very depressed
		Confused
		Drooling
		Retching
		Vomit without blood
		Vomit with blood
		Diarrhoea without blood
		Diarrhoea with blood
		Inflated/tympanic
		Haemorrhages other than from intestine
		Respiratory difficulties
		Cannot stand
		Seizures
		Unconscious
		Other (describe)
	Describe any other symptoms that the dog had	

The dog recieved the following veterinary treatement(s):	The dog was brought in dead or reported dead to a veterinarian and recieved no treatment<br>Examined and sendt home with general advice<br>Dietary advice for GI-symtpoms<br>Probiotics (e.g. ZooLac®)<br>Antibiotics<br>Fluid therapy<br>Hospitalised<br>Intensive care<br>Other (describe)
Diagnostic tests	Culturing (resistance/testing) (Results pending/Negative/Positive/Not done)<br>Parasittological testing (Results pending/Negative/Positive/Not done)<br>Parvovirus "snap-test" (Results pending/Negative/Positive/Not done)<br>Parvovirus PCR (Results pending/Negative/Positive/Not done)<br>Circovirus PCR (Results pending/Negative/Positive/Not done)<br>Coronavirus PCR (Results pending/Negative/Positive/Not done)<br>Rotavirus PCR (Results pending/Negative/Positive/Not done)<br>HCC (Canine adenovirus 1) PCR (Results pending/Negative/Positive/Not done)<br>Distempervius PCR (Results pending/Negative/Positive/Not done)<br>Leptospirosis PCR/serology (Results pending/Negative/Positive/Not done)<br>Clostridium perfringens toxins netE/netF (Results pending/Negative/Positive/Not done)
Dignostic tests	Blood test/serum - biochemistry (yes-normal/yes-abnormal/not done/results pending)<br>Blood test haemoatology (yes-normal/yes-abnormal/not done/results pending)<br>C-reactive protein (CRP) (yes-normal/yes-abnormal/not done/results pending)<br>Coagulation test (yes-normal/yes-abnormal/not done/results pending)<br>Blood gas (yes-normal/yes-abnormal/not done/results pending)
Were any pathogenic bacteria detected?	If so; what species and in what sort of sample?
Other animals than dogs in the household or on the property?	Cat<br>Rodent<br>Bird<br>Horse<br>Production animals<br>Other (describe)
Number of dogs in the household	1/2/3/4/if >4 write the number
Number of dogs in the household with vomiting or diarrhoea in the last 4 weeks	1/2/3/4/if >4 write the number
Have other animals than dogs in the house or on the property been sick with gastrointestinal symptoms in the last 4 weeks	yes/no/don't know
If yes in Q20	Describe
Has the dog been vaccinated in the last 3 years?	Parvovirus (yes/no/don't know)<br>HCC (Canine adenovirus 1) (yes/no/don't know)<br>Canine Distempervirus (yes/no/don't know)
Was the dog vaccinated when it was a puppy?	Parvovirus (yes/no/don't know)<br>HCC (Canine adenovirus 1) (yes/no/don't know)<br>Canine Distempervirus ((yes/no/don't know)
Has the dog been in contact with another dog with diarrhoea in the last 2 weeks?	yes/no/don't know
If yes in Q24	Describe

Has the dog in the two weeks before disease onset, had any of the following treatments:	Intestinal parasites
	Antibiotics
	NSAIDs (painkillers)
	Corticosteroids
	Food supplements or vitamins
	Other (describe)
Has the dog other other dogs in the the houshold been abroad in the last 2 months	yes/no/don't know
If yes in Q27, what countries?	Describe
Did the dog have a food change in the last month before disease onset?	yes/no/don't know
If yes in Q29, from which food to which food?	Describe
Did the dog have dried food in the 2 weeks before disease onset?	Yes, more or less every day/some days/perhaps/no/can't remember
Provide name/type and brand of dried food	
Did the dog have canned food in the 2 weeks before disease onset?	Yes, more or less every day/some days/perhaps/no/can't remember
Provide name/type and brand of canned food	
Did the dog have "dog sausage" in the 2 weeks before disease onset?	Yes, more or less every day/some days/perhaps/no/can't remember
Provide name/type and brand of dog sausage	
Did the dog have commercial raw food in the 2 weeks before disease onset?	Yes, more or less every day/some days/perhaps/no/can't remember
Provide name/type and brand of raw food	
Did the dog have raw rumen in the 2 weeks before disease onset?	Yes, more or less every day/some days/perhaps/no/can't remember
Provide name/type and brand of rumen	
Did the dog have dried pig ears og other products for chewing made from swine in the 2 weeks before disease onset?	Yes, more or less every day/some days/perhaps/no/can't remember
Provide name/type and brand of the product	
Did the dog have dried ox hide or other products for chewing made from ox/cow in the 2 weeks before disease onset?	Yes, more or less every day/some days/perhaps/no/can't remember
Provide name/type and brand of the product	
Did the dog have dried meat e-g- dick strips, beef strips, dried chicken or similar products in the 2 weeks before disease onset?	Yes, more or less every day/some days/perhaps/no/can't remember
Provide name/type and brand of the product	
Did the dog have othe dogs treats/goodies from a packet in the 2 weeks before disease onset?	Yes, more or less every day/some days/perhaps/no/can't remember
Provide name/type and brand of the product	
Did the dog have othe dogs treats/goodies from a pcik and mix in the 2 weeks before disease onset?	Yes, more or less every day/some days/perhaps/no/can't remember
Provide name/type and brand of the product	
Did the dog have Dentastix® or similar product in the 2 weeks before disease onset?	Yes, more or less every day/some days/perhaps/no/can't remember
Provide name/type and brand of the product	
Did the dog have any other product in the 2 weeks before disease onset?	Yes, more or less every day/some days/perhaps/no/can't remember
Provide name/type and brand of the product	

Tick the appropriate boxes relevant to the 2 weeks before disease onset:	The dog found something it ate/may have eaten outside
	The dog visited/attended a dog show or an event with other dogs
	The dog visited a veterinarian
	The dog stayed in a kennel
	The dog ate faeces from other animals
	The dog went hunting
	The dog drank from a stream/creak, river or lake
	The dog swam in a river
	The dog swam in a lake
	The dog swam in the sea
Follow up from Q 55: describe what you think the dog may have eaten/or ate:	
Follow up from Q 55: describe the dog show or event:	
Follow up from Q 55: where dog the dog og hunting and what sort of hunting was it:	
Follow up from Q 55: where did the dog og swimming, descrive the place and add municipality and postal code	
The dog was walked in the following types of places (tick relevant boxes):	In the neighbourhood (refers to postal code provided for where the dog lives)
	Other municipalities than home (home refers to postal code provided for wherethe dogs lives)
	Park or recreational area
	Urban area (dense housing)
	Farm/area with farm-animals
	Dog park
	Forest area
	By the beach/sea
	By a lake
	By a river
	Mountain
Please write the name of the areas the dog visitied in the 2 weeks before disease onset	Other municipalities than home (home refers to postal code provided for wherethe dogs lives)
	Park or recreational area
	Urban area (dense housing)
	Farm/area with farm-animals
	Dog park
	Forest area
	By the beach/sea
	By a lake
	By a river
	Mountain
Do you have any other information that you think may be relevant	Describe
